# Osteoarthrosis causing altered mental status: a case report

**DOI:** 10.1186/1752-1947-8-401

**Published:** 2014-12-03

**Authors:** Yogesh N V Reddy, Richard A Josephson

**Affiliations:** 1 Department of Cardiology, Mayo clinic, 200 1st Street SW, Rochester, MN 55905 USA; 2 Department of Cardiology, University Hospitals Case Medical Center, 11000 Euclid Ave, Cleveland, OH 44016 USA

**Keywords:** Cervical spondylosis, Hypoventilation, Phrenic neuropathy

## Abstract

**Introduction:**

Cervical spondylosis as a cause of diaphragmatic weakness is an uncommon entity and has been reported primarily in the setting of cervical spinal cord compression. Cervical spondylosis most often causes respiratory failure from cervical myelopathy and damage to the ventral horn cells at spinal cord segments C3 to C5 from where the phrenic nerve arises. The manifestations are variable but there may be evidence of upper motor neuron signs and neurological deficits in the lower extremities along with Lhermitte’s sign. Here we report a rare case of cervical spondylosis causing phrenic nerve root compression from foraminal narrowing at C3, C4 and C5, leading to lower motor neuron paralysis of the phrenic nerve and respiratory failure, in the absence of spinal cord involvement.

**Case presentation:**

An 87-year-old Caucasian man presented with recurrent episodes of hypercapnic respiratory failure and altered mental status requiring intubation. He was noted to have neurological deficits in his upper extremities with C5 radiculopathy deficits. An arterial blood gas showed a normal alveolar-arterial oxygen gradient with chronic respiratory acidosis, and pulmonary function testing showed restrictive lung mechanics with weakened neuromuscular apparatus and low maximum inspiratory and expiratory pressures. An extensive workup including electromyogram and magnetic resonance imaging showed evidence of phrenic neuropathy secondary to C3 to C5 neural foramina compression, from cervical spondylosis. He was treated conservatively with night-time bilevel positive airway pressure which rested his respiratory musculature with significant improvement.

**Conclusions:**

Cervical spondylosis leading to phrenic nerve root compression is a rare and underreported cause of chronic respiratory acidosis and must be considered in the differential diagnosis of chronic hypoventilation, particularly in the elderly. This case illustrates how a simple arterial blood gas and calculation of the alveolar-arterial oxygen gradient can help in the workup of chronic respiratory acidosis by identifying causes of hypoventilation, which are associated with a normal diffusing lung capacity and thereby a normal alveolar-arterial oxygen gradient.

## Introduction

Cervical spondylosis as a cause of diaphragmatic weakness is an uncommon entity and has been reported primarily in the setting of cervical spinal cord compression. Here we describe an unusual case of recurrent hypercapnic respiratory failure from cervical spondylosis-induced nerve root compression and diaphragmatic weakness. There was no evidence of spinal cord compression. This case illustrates how a simple arterial blood gas (ABG) and calculation of the alveolar-arterial oxygen (A-a) gradient can help in the workup of chronic respiratory acidosis by identifying causes of hypoventilation, which are associated with a normal diffusing lung capacity and thereby a normal A-a gradient.

Cervical radiculopathy resulting from nerve root compression is most often due to cervical spondylosis (cervical osteoarthritis). Osteophytes, disk protrusions, and hypertrophic facet or uncovertebral joints may compress nerve roots at the intervertebral foramina and this compression accounts for 75% of cervical radiculopathies. The underlying primary pathology is usually degenerative disease of the intervertebral discs. This is followed by reactive hyperostosis with osteophyte formation. Ischemia of the cord or roots from compression or distortion of small blood vessels may contribute to the neurologic deficit. It is important to remember however, that pain is notably absent in a significant number of cases of cervical spondylosis and may delay the diagnosis as in our patient. The pain is believed to occur only when there is dorsal root (sensory) ganglion compression [1]. The roots most commonly affected are C7 and C6. Magnetic resonance imaging (MRI) is the study of choice to define the anatomic abnormalities, but plain computed tomography is adequate to assess bony spurs, foraminal narrowing, or ossification of the posterior longitudinal ligament.

## Case presentation

An 87-year-old Caucasian man was transferred from another hospital after an episode of altered mental status secondary to hypercapnea requiring intubation. His past medical history included type 2 diabetes, stage 3 chronic kidney disease, and heart failure with preserved ejection fraction. He was a lifelong non-cigarette smoker. Per the family he was complaining of shortness of breath, weight gain and worsening leg swelling for 3 weeks, and was progressively more somnolent over the last week. On admission to the outside hospital the results of his laboratory tests were significant for a normal blood glucose of 176mg/dL, normal white blood cell count at 7000 cells/mm^3^, creatinine of 1.6mg/dL (at his baseline), elevated brain natriuretic peptide at 778pg/mL and negative toxicology screen. He had no history of opioid or benzodiazepine usage. His ABG showed acute on chronic hypercapnea with a pH of 7.22, partial pressure of carbon dioxide (pCO_2_) of 83mmHg and bicarbonate (HCO_3_) of 36mmol/L requiring intubation explaining his altered mental status. He was noted to be in congestive cardiac failure and was given diuretics and eventually he was extubated. He was transferred to our institution on nightly bilevel positive airway pressure (bipap) for his unexplained hypercapnic failure. His only complaint on transfer was his chronic two-pillow orthopnea.

On arrival he was in no acute distress with a heart rate of 74, respiratory rate of 20, blood pressure of 107/65mmHg, and oxygen saturation of 98% on 2L nasal cannula. A cardiovascular and respiratory system examination was unrevealing with normal jugular venous pressure and normal cardiac examination. There was paradoxical abdominal motion with the abdominal wall moving inwards on inspiration. A neurologic examination revealed 3/5 strength in his left deltoid and decreased biceps reflexes bilaterally. Power, tone and reflexes were normal in both his lower limbs. He also had severe osteoarthrosis of distal and proximal interphalangeal joints bilaterally with Heberden’s nodes.

**Figure 1:**
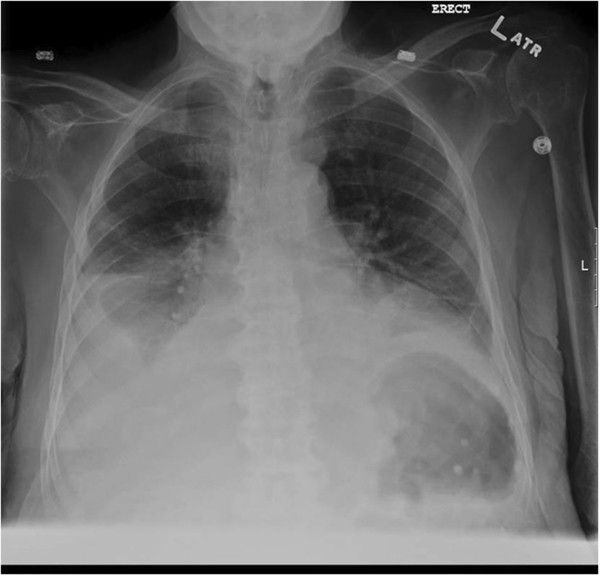
**Chest X-ray showing elevated hemidiaphragms bilaterally with pleural effusions, perihilar edema and cardiomegaly.**

His ABG on room air showed a partial pressure of oxygen of 64mmHg, pCO_2_ of 53mmHg, HCO_3_ of 28mmol/L and a pH of 7.33 with a normal A-a gradient of 18. This was consistent with chronic respiratory acidosis secondary to hypoventilation, given the normal A-a gradient. A chest X-ray showed elevated hemidiaphragms bilaterally (Figure 1). Pulmonary function testing showed a restrictive pattern with a normal ratio of forced expiratory volume in 1 second (FEV1) to forced vital capacity (FVC) of 0.79 (114% predicted), with FEV1 and FVC 49 and 42% of predicted respectively. There were also significantly decreased maximum inspiratory and maximum expiratory pressures of 27.2 and 31.4% predicted; and a decrease in FVC of 22% from the sitting to supine position suggestive of neuromuscular weakness (>20%). The diffusing capacity was normal suggesting a chest wall or neuromuscular cause of restrictive lung mechanics.

Given his abnormal neurologic examination with left deltoid weakness and decreased biceps reflexes, our differential included respiratory neuromuscular weakness from cervical spondylosis and phrenic nerve root compression (given the C5 neurological deficits); or possible cervical myelopathy at C5/C6. The absent biceps reflex (C5 and C6) raised suspicion for myelopathy at the C5 to C6 level although this is typically associated with a brisk triceps reflex (C7). The lack of neurological findings in his lower extremities also argued against myelopathy, but given the potential variable manifestations of cervical myelopathy, an electromyogram (EMG) and nerve conduction study (NCS) were performed. The EMG and NCS revealed decreased amplitude in the phrenic nerves bilaterally and radiculopathy in his upper limbs, suggestive of nerve root compression at the cervical foraminal level. There was no evidence of amyotrophic lateral sclerosis or myopathy on EMG/NCS and serum creatine phosphokinase was normal. A cervical MRI was performed and showed severe bilateral foraminal narrowing at C3, C4 and C5 with no evidence of myelopathy, confirming the diagnosis of phrenic nerve root compression from cervical spondylosis as the cause of hypoventilation. When he developed pulmonary edema, the resultant decreased lung compliance placed an increased respiratory load on his weakened neuromuscular apparatus leading to worsening hypercapnea and resultant metabolic encephalopathy with altered mental status. Carbon dioxide, being readily diffusible across the blood–brain barrier is a well-known cause of encephalopathy in the setting of hypercapnea.

He was offered surgical decompression of cervical motor roots or possible diaphragmatic pacing. Given his age, he opted for conservative management with nightly bipap to rest his respiratory muscles at night. He showed significant improvement with night-time bipap and felt less fatigued during the day, probably from the rest provided to his respiratory neuromuscular apparatus. Following discharge he remained stable but gradually became less compliant with bipap. He had four admissions in 2 years with hypercapnic respiratory failure in the setting of pulmonary edema from heart failure. Pulmonary edema and the resultant decreased lung compliance placed an increased respiratory load on his weakened neuromuscular apparatus. As expected he fatigued more easily with his weakened neuromusculature leading to hypoventilation and worsening hypercapnea. He required continuous bipap while hospitalized until diuresis improved chest wall compliance and respiratory mechanics. He continued to refuse invasive therapies and was managed conservatively with nightly bipap. He developed evidence of right ventricular failure and pulmonary hypertension on echo from a combination of pulmonary venous hypertension from heart failure and chronic hypoxia from hypoventilation secondary to his neuromuscular weakness. Given his recurrent episodes of respiratory failure and worsening heart failure he was eventually transitioned to hospice care.

## Discussion

The phrenic nerve arises from the anterior rami of C3 to C5 spinal nerves. While phrenic nerve dysfunction has been previously reported following myelopathy and anterior horn cell damage from cervical spondylosis, our patient had no evidence of spinal cord damage and the phrenic neuropathy was from bilateral nerve root compression at C3 to C5 [2, 3]. Bilateral phrenic neuropathy from cervical spondylosis is a rare cause of diaphragmatic weakness and respiratory failure with only sporadic case reports [4]. The low incidence may reflect an underreporting of cases. Similar to cervical radiculopathy, the signs of cervical spondylotic myelopathy are variable. If present, cervical spinal cord involvement may be indicated by weakness or stiffness in the legs, or unsteadiness of gait. Some patients may exhibit Lhermitte’s sign which is an electrical sensation elicited by neck flexion that radiates down the spine from the neck.

Bilateral diaphragmatic paralysis can occur from motor neuron disease, myopathy, acid maltase deficiency, lupus (shrinking lung syndrome), end-stage renal disease, hypo or hyperthyroidism and malnutrition [5]. Diabetes by itself can cause phrenic nerve neuropathy and this has been reported in the literature [6]. Although our patient was diabetic, it was felt less likely that the neuropathy was related to diabetes given the neural foraminal compression and nerve impingement noted on MRI at C3 to C5. We cannot exclude, however, that diabetic neuropathy potentially was a contributor to some of the phrenic nerve dysfunction. But with prominent compression noted on the MRI it was felt that compression was the major determinant of phrenic nerve dysfunction.

Even after diuresis our patient had persistent orthopnea, which occurs in bilateral paralysis because the abdominal contents displace the diaphragm into the thorax when supine, which results in a significant (>500mL) decrease in vital capacity and oxygen saturation. Fluoroscopy is not reliable in this situation because the flaccid diaphragm may lag behind the ribcage expansion when accessory muscles contract, thus giving the impression of diaphragmatic contraction. This limitation is not seen in unilateral diaphragmatic paralysis, where the sniff fluoroscopy test is positive in over 90% of cases. In cervical radiculopathy many of the accessory muscles of respiration can also become weak from cervical nerve root compression, and contribute to the already increased load on these muscles from the diaphragmatic dysfunction. Intermittent ventilatory support can help with respiratory muscle fatigue in this setting, and this was the rationale behind him being discharged on nocturnal bipap.

## Conclusions

Cervical spondylosis-induced phrenic nerve compression should be considered in the differential diagnosis of respiratory failure with a normal A-a gradient, especially when there is evidence of cervical radiculopathy on neurological examination. An ABG on room air with calculation of the A-a gradient, can help in the evaluation of unexplained hypoxemia and hypercapnea as a measure of diffusion across the capillary membrane. This is also much more practical to perform in patients in ventilatory failure or respiratory distress, than formal pulmonary function testing with diffusing lung capacity. The formula for the A-a gradient = (pIO_2_–1.25(PaCO_2_))–PaO_2_. A normal A-a gradient implies that hypoxemia is either from hypoventilation or from decreased atmospheric pressure (as in high altitude) with a normal diffusion membrane in the lung. However, the A-a gradient may be mildly elevated in those patients that develop significant basal atelectasis and ventilation/perfusion mismatch and hence a high index of suspicion is required for this rare but potentially reversible cause of respiratory failure.

## Consent

Written informed consent was obtained from the patient for publication of this case report and accompanying images. A copy of the written consent is available for review by the Editor-in-Chief of this journal.

## Abbreviations

A-a: Alveolar-arterial oxygen
ABG: Arterial blood gas
bipap: Bilevel positive airway pressure
EMG: Electromyogram
FEV1: Forced expiratory volume in 1 second
FVC: Forced vital capacity
HCO_3_: Bicarbonate
MRI: Magnetic resonance imaging
NCS: Nerve conduction study
PaCO_2_: Partial pressure of carbon dioxide in arterial blood
PaO_2_: Partial pressure of oxygen in arterial blood
pCO_2_: Partial pressure of carbon dioxide
pIO_2_: Partial pressure of inspired oxygen.
